# Exploring the Hidden Complexity: Entropy Analysis in Pulse Oximetry of Female Athletes

**DOI:** 10.3390/bios14010052

**Published:** 2024-01-19

**Authors:** Ana M. Cabanas, Macarena Fuentes-Guajardo, Nicolas Sáez, Davidson D. Catalán, Patricio O. Collao-Caiconte, Pilar Martín-Escudero

**Affiliations:** 1 Departamento de Física, Universidad de Tarapacá, Arica 1010069, Chile; nvalderramas@academicos.uta.cl (N.S.); david.catalan.ayala@alumnos.uta.cl (D.D.C.); 2 Departamento de Tecnología Médica, Universidad de Tarapacá, Arica 1010069, Chile; mafuentesg@academicos.uta.cl; 3 Dirección de Gestión Digital y Transparencia, Universidad de Tarapacá, Arica 1000007, Chile; pcollaoc@gestion.uta.cl; 4 Medical School of Sport Medicine, Faculty of Medicine, Universidad Complutense de Madrid, 28040 Madrid, Spain; pmartinescudero@med.ucm.es

**Keywords:** pulse oximeter, approximate entropy, sample entropy, *VO*_2,*max*_, women’s response to exercise

## Abstract

This study examines the relationship between physiological complexity, as measured by Approximate Entropy (ApEn) and Sample Entropy (SampEn), and fitness levels in female athletes. Our focus is on their association with maximal oxygen consumption ( $VO_{2,max}$ ). Our findings reveal a complex relationship between entropy metrics and fitness levels, indicating that higher fitness typically, though not invariably, correlates with greater entropy in physiological time series data; however, this is not consistent for all individuals. For Heart Rate (HR), entropy measures suggest stable patterns across fitness categories, while pulse oximetry ( $SpO_{2}$ ) data shows greater variability. For instance, the medium fitness group displayed an ApEn(HR) = 0.57±0.13 with a coefficient of variation (CV) of 22.17 and ApEn( $SpO_{2}$ ) = 0.96±0.49 with a CV of 46.08%, compared to the excellent fitness group with ApEn(HR) = 0.60±0.09 with a CV of 15.19% and ApEn( $SpO_{2}$ ) = 0.85±0.42 with a CV of 49.46%, suggesting broader physiological responses among more fit individuals. The larger standard deviations and CVs for $SpO_{2}$ entropy may indicate the body’s proficient oxygen utilization at higher levels of physical demand. Our findings advocate for combining entropy metrics with wearable sensor technology for improved biomedical analysis and personalized healthcare.

## 1. Introduction

In recent years, there has been a growing interest in understanding the physiological responses of women to exercise, particularly regarding variations in oxygen saturation [1]. Notably, women have been observed to experience a premature decrease in oxygen saturation during maximal exercise, occurring at lower oxygen intakes than in men [2]. This early decline has sparked discussions about its underlying causes, with some researchers suggesting that healthy, active women may encounter exercise-induced arterial hypoxia due to anatomical differences in lung structure and capacity that impact oxygen diffusion [3]. However, recent studies have shifted the focus to the role of oxygen desaturation in limiting the achievement of peak maximum oxygen uptake ( $VO_{2,max}$ ) levels, suggesting that factors beyond lung size or capacity are at play [4].

The evolution of wearable sensors, particularly those tracking essential metrics such as heart rate (HR) and oxygen saturation ( $SpO_{2}$ ) through photoplethysmography (PPG) [5], stands as a watershed moment in comprehending the effects of exercise and fine-tuning training programs [6]. Among these advancements, pulse oximetry has emerged as a pivotal non-invasive technique, indispensable for evaluating oxygenation levels during physical exertion. Its ability to provide continuous monitoring of peripheral oxygen saturation changes delivers invaluable real-time insights into an athlete’s oxygenation status, significantly enhancing our grasp of physiological responses during exertion. Despite its immense potential, pulse oximetry encounters limitations, including susceptibility to movement artifacts [7], challenges associated with varying skin pigmentation [8,9,10], and other technical constraints [11,12,13]. Nonetheless, the integration of these wearable biofluid monitoring sensors and devices, especially pulse oximetry, has remarkably enriched our understanding of how the human body reacts to physical stress, emphasizing the crucial role these technologies play in optimizing athletic performance and overall health.

Maintaining optimal oxygen levels is critical for athletes’ performance, recovery, and health. Low oxygen saturation, particularly during intense workouts or at high altitudes, can lead to fatigue, decreased performance, and altitude sickness [14]. Continuous monitoring of these levels enables informed decisions about training and recovery strategies, optimizing performance through tailored approaches [15].

Recent studies emphasize the variability patterns in $SpO_{2}$ signals, providing insights into respiratory control and breathlessness sensation under hypoxic conditions [16]. Variations in $SpO_{2}$ correlate with breathlessness perception, reflecting the complex interplay among various respiratory indicators [17,18].

Assessing regularity within dynamical systems spans various scientific and engineering disciplines, with a growing importance in understanding complexity within biological datasets [19,20]. Methods like Kolmogorov complexity [21], $\frac{C1}{C2}$ complexity measure [21], and entropy [22] have been developed to quantify complexity in time series data.

In biomedicine, Approximate Entropy (ApEn) [23] and Sample Entropy (SampEn) [24] are crucial for analyzing physiological time series, aiding in pattern identification and anomaly detection within these signals [25,26,27]. Entropy methods offer advantages over traditional methods, improving diagnostic systems, particularly in heart disorders [28,29]. They also provide insights into health aspects like heart rate variability (HRV) [30,31], reflecting autonomic nervous system health, with low entropy values indicating potential pathological conditions or diminished regulation [32]. Similarly, entropy of electroencephalogram signals can unveil brain function insights, correlating complexity alterations with neurological conditions [33].

Beyond diagnostics, entropy measures can be used to distinguish expert athletic performances [34] and between fallers and non-fallers [35], and identify various health aspects such as fall risks [35], effects of aging on gait [36,37], detect physical fatigue [38], respiratory dysfunctions like sleep apnea [39], stress responses [40], or report valuable information to running training methods [41] among others.

Pulse oximetry and heart rate data are key indicators of cardiovascular and respiratory health, ripe for entropy-based evaluations. For female athletes, these metrics are critical not only for assessing well-being but also for understanding performance capabilities and resilience [42].

The significance of pulse oximetry in sports lies in its ability to uncover vital insights into an individual’s blood oxygen-carrying capacity, particularly crucial during intense physical exertion. Essential in assessing athletic prowess, especially in endurance sports, is the $VO_{2,max}$ parameter, signifying the peak of oxygen consumption during exercise [43]. This metric not only correlates with cardiovascular health and aerobic endurance but also holds implications for longevity, being a robust predictor of mortality and functional capacity [44]. Additionally, exploring age-related physiological changes through exercise emphasizes its pivotal role in enhancing life expectancy and overall health [45].

This work delves into the intricacies of Approximate Entropy (ApEn) and Sample Entropy (SampEn), with a focus on their application to time-series data derived from pulse oximetry and heart rate measurements in female athletes. Our mission is to offer a robust and consistent statistical measure of system complexity, to clarify the nuances that differentiate these entropy measures and their calculation parameters, and to elucidate their relationship with maximum oxygen uptake $VO_{2,max}$ . The continuous monitoring of oxygen levels and heart rate is crucial for optimizing training and recovery strategies, as these metrics directly affect athletic performance. This highlights the need for such detailed analysis.

## 2. Methods

### 2.1. Protocol and Testing Procedure

The study protocol comprised an anamnesis that encompassed medical history and sports training, along with physical examinations. These examinations, conducted prior to obtaining informed consent, included cardiovascular and pulmonary auscultation, blood pressure measurement, and the recording of weight and height to calculate the Body Mass Index (BMI). Subsequently, a maximal treadmill incremental exercise test was performed. This test entailed continuous electrocardiographic (ECG) recording and ergospirometry using a breath-by-breath gas analyzer (Sensor Medics Vmax Cardiopulmonary Sanro). Additionally, pulse oximetry monitoring was uninterrupted during the warm-up, maximal exercise, and recovery phases. For this purpose, a commercial pulse oximeter, the Pulsox-3i Minolta, Konica Minolta, Tokio, Japan, was used.

To ensure data synchronization, the ergospirometry and oximeter were aligned, with readings taken every second throughout the stress test. Prior to the treadmill assessment, a forced spirometry was executed, and the pulse oximeter was calibrated for one minute post-cleaning for precise oxygen saturation measurements. Concurrently, heart rates were monitored via ECG and oximeter, and blood pressure was tracked continuously.

The stress test commenced on a treadmill ergometer (HP Cosmos QUASAR 4.0), with an initial one-minute standing baseline data collection. The warm-up consisted of walking at 6 km/h with a 1% incline for 2 min, progressing to a running phase at 8 km/h on the same gradient. As the athletes reached peak effort, they disengaged from the treadmill. The effort increased incrementally: upon reaching 14 km/h, the incline was raised to 3%, and thereafter, speed was increased by 2 km/h every 2 min until exhaustion. During a 2 min active recovery at 8 km/h with a 0% incline, ECG readings were taken every 10 s, averaging the last eight heartbeats. Step rate (SR) was manually calculated during consistent running phases. Post-exercise, blood pressure measurements were taken at 3 and 5 min into recovery. All athletes adhered to this protocol, with individual variations only in the maximal effort achieved [4].

All tests were conducted at the Physiology Laboratory of the Professional School of Sports Medicine at the Faculty of Medicine, Universidad Complutense de Madrid, Spain. Participants provided written consent after being informed about the study’s procedures and associated risks. The inclusion criteria for participants were:

- Females aged 13 to 55.
- Engaged in regular competitive sports practice at national and regional tournaments for a minimum of 2 years prior to the study.
- Training frequency of 2 to 4 times a week, with sessions lasting between 1 to 3 h. Continued their sports practice up until the day preceding the study.
- No reported respiratory or cardiac diseases and exhibited normal spirometric values. Underwent an evaluation for cardiovascular health prior to the study.

Table 1 presents the anthropometric and clinical details of the twenty-seven active and healthy female volunteers, including age, size, weight, body mass index (BMI), maximum heart rate $HR_{max}$ and maximum oxygen uptake ( $VO_{2,max}$ ). The values presented are averages, accompanied by their respective standard deviations, to provide an understanding of the variability within the data.

Participants were stratified into three fitness categories based on their maximal oxygen uptake ( $VO_{2,max}$ ), as indicated in Table 2 [4]. The excellent fitness group, constituting 40.74% of the participants, had a mean $VO_{2,max}$ of 55.99 mL/kg/min, with values ranging from 50.90 to 66.20 mL/kg/min. The good fitness group, also 40.74%, had a mean of 46.75 mL/kg/min, spanning from 41.00 to 50.00 mL/kg/min. The medium fitness group made up 18.52% with a closer range of $VO_{2,max}$ , having a mean of 38.10 mL/kg/min and low variability, as indicated by a standard deviation of 0.55.

### 2.2. Entropy-Based Regularity Assessment of Time Series Data

Entropy, a fundamental concept in thermodynamics, measures the disorder within a closed system and is crucial in assessing complexity within nonlinear dynamical systems. This concept is particularly valuable for analyzing time series due to its flexible approach to probability distribution [23]. Shannon’s entropy and conditional entropy are key metrics for quantifying the amount and rate of information generation, respectively [19]. These metrics form the foundation for other entropy measures designed to investigate time series intricacies. Entropy provides researchers with the ability to quantify complexity even in short datasets, enhancing the significance of experimental comparisons with control groups.

Pincus introduced Approximate Entropy (ApEn), a widely-used metric that measures regularity, quantifying complexity levels within a time series [23]. ApEn assesses system complexity akin to entropy, making it suitable for analyzing clinical cardiovascular and other time series data. Additionally, Sample Entropy (SampEn), introduced by Richman and Moorman [24], aligns more closely with theoretical expectations compared to ApEn across different conditions [24]. SampEn’s increased precision makes it particularly valuable for scrutinizing experimental clinical cardiovascular and other biological time series data.

For our study, time-series data related to pulse oximetry and heart rate from the twenty-seven physically active and healthy female participants were collected. To quantify the regularity and complexity of our time-series data, we employed two entropy-based metrics: Approximate Entropy and Sample Entropy.

ApEn measures the unpredictability of fluctuations within a time-series dataset. It has been widely adopted in biomedical domains due to its ability to handle short and noisy datasets. ApEn is robust against noise, applicable to both stochastic and deterministic processes, and yields non-negative values indicative of complexity [26,44].

Given a time-series data of length *N*, u(i),u(2),…,u(N) , the following steps outline its computation:

1. Fix parameters: *m* (pattern length) and *r* (similarity criterion).
2. Form *N* − *m* + 1 vector of length m from the time series $x_{m}\left(i\right)$ . The distances between them is: $d\left[x\left(i\right),x\left(j\right)\right]=max_{k}(|\left(i+k\right)-u\left(j+k\right)|)$ with 0≤k≤m−1
3. For each vector, count the number of vectors that are similar to it within a tolerance r. $C_{i}^{m}\left(r\right)=($ number of j≤N−m+1 such that d[x(i),x(j)]≤r)/(N−M+1).
4. Compute the regularity measure for patterns of length m as:
$$\varphi^{m}\left(r\right)=\frac{1}{N-m+1}\sum_{i=1}^{N-m+1}logC_{i}^{m}\left(r\right)$$
5. The statistical estimator of the *ApEn(m, r, N)* is then defined as
$$ApEn\left(m,r,N\right)\left(u\right)=\varphi^{m}\left(r\right)-\varphi^{m+1}\left(r\right)$$

SampEn, an evolution of ApEn, was crafted to be less reliant on the length of the time series and to exhibit greater consistency [24]. It addresses biases and inconsistencies inherent to ApEn. Notably, SampEn’s computation excludes self-matches, making it a more unbiased estimator of system complexity. Given an identical time series, the computation unfolds as follows:

1. Similar to the steps in ApEn, begin with a time series of length N and construct vectors.
2. However, in counting the number of matches, do not include self-matches (i.e., exclude the case *j* = *i*).
3. Define regularity measures for sequences of length m as:
$$B^{m}\left(r\right)=\frac{1}{N-m}\sum_{i=1}^{N-m}C_{i}^{m}\left(r\right)$$
  and
$$A^{m}\left(r\right)=\frac{1}{N-m-1}\sum_{i=1}^{N-m-1}C_{i}^{m+1}\left(r\right)$$
4. Compute *SampEn(m, r, N)(u)* as:
$$SampEn\left(m,r,N\right)=-ln\frac{A^{m}\left(r\right)}{B^{m}\left(r\right)}$$

In essence, both ApEn and SampEn gauge the regularity or unpredictability of time-series data. However, SampEn’s intentional exclusion of self-matches endows it with a more refined approach. This nuanced counting technique typically results in SampEn delivering more consistent and trustworthy outcomes compared to ApEn [46].

To investigate the influence of parameters on entropy calculations, diverse combinations of ApEn and SampEn parameters were used for the time series data. These included *m* (data comparison length) with values of 1, 2, and 3, and *r* (sensitivity criterion) set at 0.1, 0.15, 0.20, and 0.25 times the standard deviation of the entire time series. The parameter *N* (data length) signifies the total number of steps in the series. Herein, *m* delineates the steps included in a sequence compasrison, while *r* specifies the permissible variance in step lengths. For instance, at m=2 , two sequential steps are juxtaposed, and with r=0.2SD , step lengths are deemed similar if they diverge by less than 20% of the series’ comprehensive standard deviation. It is worth noting that a pragmatic strategy involves defining the tolerance as r=0.2SD , where SD epitomizes the standard deviation of the dataset [27]. This approach eases comparisons between datasets with disparate amplitudes [25]. For this investigation, every time series underwent normalization to achieve an SD of 1.

## 3. Results

**Figure 1:**
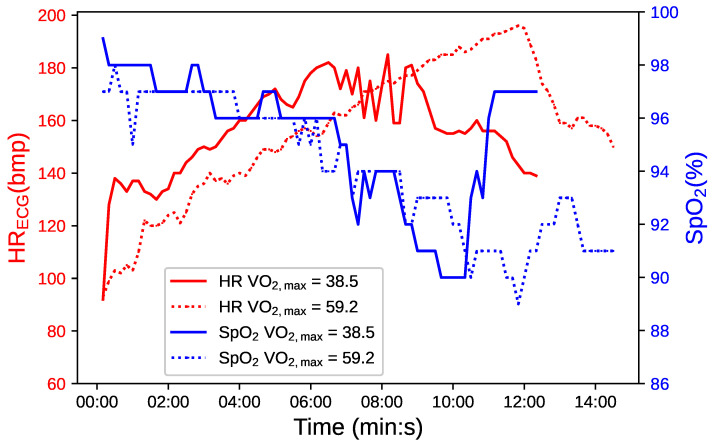
Comparative temporal evolution of $SpO_{2}$ in blue and HR in red for two athletes with varying fitness levels, distinguished by continuous and dotted lines.

Figure 1 presents a comparative analysis of cardiovascular and respiratory responses during exercise between two athletes with different fitness levels. The graph utilizes a dual-y-axis format to display heart rate (HR) in red and oxygen saturation ( $SpO_{2}$ ) in blue over the course of the exercise test, marked in minutes and seconds on the x-axis.

The continuous line correlates with an athlete with medium fitness ( $VO_{2,max}$ = 38.5 mL/(kg·min), and the dotted line with an athlete of excellent fitness ( $VO_{2,max}$ = 59.2 mL/(kg·min). The latter demonstrates a longer duration of exercise, indicative of superior cardiovascular and respiratory endurance. Interestingly, this athlete also experiences more pronounced $SpO_{2}$ fluctuations. The notable $SpO_{2}$ variability observed in the athlete with higher $VO_{2,max}$ reflects this efficient oxygen utilization, which is particularly evident during periods of intense physical activity [47,48].

Enhanced oxidative capability in the muscles of a highly fit athlete facilitates operation at lower partial pressures of oxygen ( $PO_{2}$ ), leading to steeper oxyhemoglobin dissociation curves and more efficient oxygen delivery [49].

These observations suggest that greater fluctuations in $SpO_{2}$ may signify an advanced level of physiological adaptation and not necessarily a decrease in cardiorespiratory function. This recontextualizes the interpretation of $SpO_{2}$ drops, proposing that, for athletes with high cardiovascular efficiency, such drops are a characteristic of robust oxygen utilization rather than a sign of impairment. [4]

**Figure 2:**
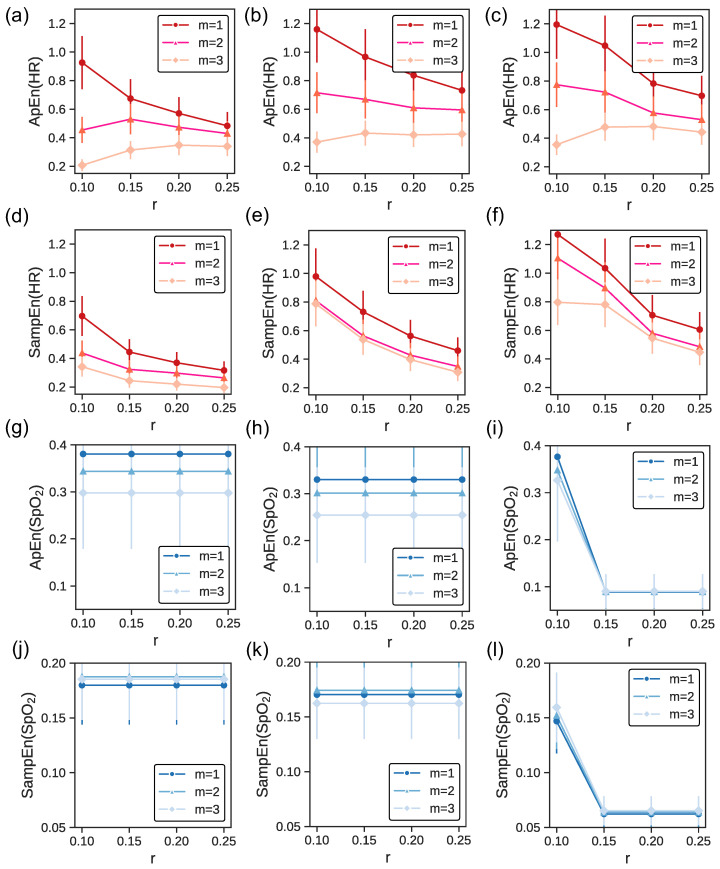
ApEn and SampEn for heart rate (in red) and $SpO_{2}$ (in blue) across fitness levels based on $VO_{2,max}$ . The first (panels (**a**,**d**,**g**,**j**)) second (panels (**b**,**e**,**h**,**k**)), and third (panels (**c**,**f**,**i**,**l**)) columns correspond to an athlete of medium, good, and excellent fitness condition, respectively. The errorbars show the SD for each fitness category.

Our study also examined the impact of varying *m* (embedding dimension) and *r* on the entropy calculations for HR and time series, exploring how these parameters affect the measurement of physiological complexity. Figure 2 displays ApEn and SampEn metrics for three athletes, each representing a distinct level of cardiovascular fitness. The variation in entropy measures is visualized for both heart rate (HR) and pulse oximetry ( $SpO_{2}$ ), with HR represented in shades of red and $SpO_{2}$ in shades of blue. Error bars illustrate the standard deviation (SD) within each category, highlighting the variability of entropy values, which serves as an indicator of the complexity and irregularity of the physiological time series data. These visualizations underscore the relationship between an athlete’s fitness level and the corresponding entropy metrics, with the variability suggested to reflect individual physiological adaptations and responses to physical stress.

The first column presents data for an athlete with a medium level of fitness, characterized by a maximal oxygen uptake $VO_{2,max}$ of less than 40 mL/kg/min. For both HR and $SpO_{2}$ , ApEn and SampEn values remain relatively consistent across different *r* values, irrespective of *m* values. This uniformity implies that for athletes at this fitness level, increasing the dimensionality (through *m*) does not drastically alter entropy estimates across various threshold values (*r*). Lower entropy values, indicating more regularity and reduced complexity, suggest that athletes of medium fitness might exhibit more predictable and consistent physiological responses.

The second column details an athlete in good physical condition ( $VO_{2,max}$ between 40 and 50 mL/kg/min). The ApEn(HR) and SampEn(HR) values here are higher than in panel (a) and d), respectively. This elevation in entropy suggests that as fitness enhances, there is an emergence of more intricate physiological patterns. However, in the case of $SpO_{2}$ the values of ApEn( $SpO_{2}$ ) and SamApEn( $SpO_{2}$ ) are marginally lower than in panel (g) and (j). Such complexity might arise from the adaptive capabilities of the cardiovascular and respiratory systems, conditioned to accommodate diverse physical challenges. Furthermore, the ApEn and SampEn values for both HR and $SpO_{2}$ remain relatively steady across different *r* and *m* values. This further corroborates that for athletes with good fitness levels, increasing the dimensionality does not substantially modify entropy estimates across different threshold values (*r*).

The third column features an athlete with excellent fitness, exhibiting a $VO_{2,max}$ of over 50 mL/kg/min. As before, ApEn(HR) and SampEn(HR) values here are higher than in panel (a) and (b), and (d) and (e) respectively. A marked variability in entropy values can be observed across different *r* values, especially when m=2 . This fluctuation might allude to the intricate physiological responses and adaptations in athletes with advanced fitness levels. Such athletes possibly have a cardiovascular system that is highly adaptable, and primed for dynamic responses to varied physiological demands.

Across the panels, SampEn values consistently appear lower than ApEn values for both HR and $SpO_{2}$ . ApEn can be biased, especially for short datasets, as it counts self-matches, leading to higher values. In contrast, SampEn eliminates this bias by excluding self-matches, often resulting in lower values [24].

When examining the complexity and regularity of physiological time series using ApEn and SampEn, the choice of parameters *m* and *r* is crucial. In our case, the choice of m=2 and r=0.2SD is supported by both empirical and theoretical considerations [1, 2]. Statistical analysis has shown that for m=2 , the entropy provides a stable measure across different conditions and subjects. Additionally, using m=2 is computationally efficient, as the computational demands increase exponentially with the embedding dimension. The choice of r=0.2SD is based on examining patterns that deviate by 20% of the standard deviation of the time series. This threshold is also consistent with many previous studies on physiological time series [32,50,51], providing a balance between sensitivity, reliability, computational efficiency, and discriminative power. Moreover, such consistency allows for improved comparability across studies and conditions [27].

Table 3 presents a comparative analysis of Approximate Entropy and Sample Entropy across different fitness levels for athletes, with measures for m=2 and r=0.2SD . The values are reported as means ( $\bar{X}$ ) with standard deviations (SD) and the coefficient of variation (CV) is included to assess variability and consistency within the data.

ApEn(HR) values marginally increase from the Medium to Excellent fitness categories, with the most notable precision observed in the Excellent category, exhibiting the lowest CV of 15.19%. The Medium fitness category shows an ApEn(HR) = 0.57±0.13 , denoting moderate variability. In contrast, ApEn values for blood oxygen saturation ( $SpO_{2}$ ) demonstrate a decrease with higher fitness, hinting at more stable $SpO_{2}$ patterns in fitter athletes and higher variability denoted by higher CVs. SampEn(HR) positively correlates with fitness levels, underscoring the link between heightened fitness and increased heart rate complexity. The SampEn( $SpO_{2}$ ) values are relatively low for all fitness levels but show a subtle rise with fitness enhancements, indicating a slight increase in complexity among the most fit athletes. For instance, Medium fitness individuals exhibit SampEn(HR) = 0.33±0.08 and an SampEn( $SpO_{2}$ ) = 0.19±0.04 , suggesting a more predictable $SpO_{2}$ pattern compared to ApEn( $SpO_{2}$ ) but also less variability with a lower CV.

Good fitness athletes have an ApEn(HR) = 0.56±0.09 , similar to the Medium group, with a marginally lower ApEn( $SpO_{2}$ ) = 0.90±0.43 . Their HR SampEn is 0.36±0.12 , and SampEn( $SpO_{2}$ ) = 0.22±0.10 , indicating a slight increment in complexity from the Medium fitness level.

The Excellent fitness group shows the highest ApEn(HR) = 0.60±0.09 , indicating significant variability. The ApEn( $SpO_{2}$ ) for this group is 0.85±0.42 , slightly reduced from the Good category, which may point to a pattern of more regularity with advanced fitness levels. However, their SampEn(HR) reaches 0.40±0.15 , the peak among the groups, and the SampEn( $SpO_{2}$ ) remains consistent with the Good group at 0.22±0.12 , with a slightly higher SD.

Overall, the data suggest an association between higher fitness and more complex heart rate patterns as indicated by both ApEn and SampEn. ApEn( $SpO_{2}$ ) implies more uniformity with improved fitness, while SampEn suggests a minimal increase in complexity. Despite this, the large standard deviations, especially noted in ApEn( $SpO_{2}$ ) values, call for a cautious interpretation of the results, and no definitive conclusions can be drawn regarding the entropy levels of $SpO_{2}$ .

We observed that while ApEn typically yields higher entropy values compared to SampEn, this does not necessarily reflect a lack of consistency. Rather, the differences between ApEn and SampEn can be attributed to their distinct computational approaches, with ApEn including self-matches and SampEn excluding them. Our data show that ApEn exhibits smaller standard deviation values than SampEn for heart rate variability, suggesting a degree of consistency within this context. Conversely, for $SpO_{2}$ variability, ApEn presents with higher standard deviation values. These observations underscore the importance of context when interpreting the results of entropy measures and reinforce the need for careful consideration in selecting the most suitable metric for a given dataset, particularly when dealing with shorter time series where the exclusion of self-matches by SampEn could be especially pertinent.

One potential cause for the variability in $SpO_{2}$ measurements could be artifacts introduced by the devices themselves [11,13]. Optimizing algorithms within pulse oximeters, designed to enhance the signal-to-noise ratio, may inadvertently alter the waveform being measured. Such alterations can lead to inaccuracies in entropy calculations, as they depend heavily on the fidelity of the signal. Moreover, the act of measuring oxygen saturation is notably more challenging during periods of intense physical activity [4]. Factors such as motion artifacts—brought on by the increased movement of the subject—or physiological changes like fluctuations in peripheral blood flow can significantly distort the readings [52]. These distortions are critical to consider, as they can mimic or mask true physiological responses, thereby affecting the entropy analysis.

In contrast, HR measurements are generally less susceptible to such artifacts and confounders. As evidenced by the data, HR entropy demonstrates less variation within fitness categories (e.g., a standard deviation of 0.09 in both ’Good’ and ’Excellent’ conditions for ApEn), suggesting a more reliable capture of cardiovascular complexity. This disparity in measurement stability between HR and $SpO_{2}$ is crucial, especially when leveraging entropy as a metric for assessing physiological complexity.

**Figure 3:**
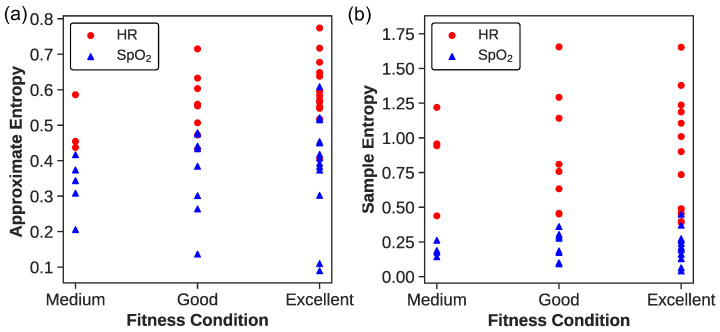
(**a**) ApEn and (**b**) SampEn for m=2 and r=0.2SD . The red dots correspond to the HR data, while the blue triangles correspond to the $SpO_{2}$ data.

For a deeper insight, Figure 3 illustrates the values of ApEn and SampEn with the parameters set for m=2 and r=0.2 . Each dot represents an athlete’s entropy values, with red dots denoting heart rate data and blue triangles indicating peripheral oxygen saturation ( $SpO_{2}$ ) data. Analyzing these entropy measures across fitness categories reveals a trend suggesting that higher fitness levels correlate with increased complexity in physiological time series data, although the relationship is not strictly linear.

For ApEn(HR), athletes in the “Excellent” category exhibit a wide range of values between approximately 0.404 and 0.774, indicating diverse heart rate complexities. The ”Good” and “Medium” categories show overlapping ranges, from about 0.433 to 0.716 and 0.437 to 0.706, respectively, with the “Medium” category representing the lower end of the spectrum. This suggests that while the fitness level may generally be associated with heart rate complexity, the distinction between categories is not clear-cut.

The ApEn( $SpO_{2}$ ) values present a similar pattern of variability and overlap. Athletes labeled “Excellent” range from 0.089 to 0.608, revealing significant diversity in the complexity of $SpO_{2}$ signals even at high fitness levels. The ”Good” category spans from 0.136 to 0.479, and the “Medium” from approximately 0.205 to 0.416. These findings highlight that individual physiological differences may play a substantial role in the complexity of $SpO_{2}$ patterns, beyond the influence of fitness level alone.

The SampEn(HR) data offers insight into the complexity and variability of heart rate dynamics within each fitness category. Athletes in the Excellent category exhibit a SampEn range from 0.390 to 1.653, indicating a wide spectrum of heart rate complexities. Similarly, the Good category displays a range from 0.398 to 1.655, and the Medium category from 0.438 to 1.792, both reflecting substantial variability. The distribution of SampEn values, particularly the highest observed value of 1.792 in the Medium group, emphasizes that individual variations can defy the general expectations based on fitness levels alone. This suggests that factors beyond fitness, possibly including intrinsic physiological differences or measurement artifacts, may influence the complexity metrics derived from heart rate data.

The SampEn( $SpO_{2}$ ) analysis reveals an intriguing trend within the Excellent category, which exhibits SampEn values extending from 0.041 to 0.449. Not only does this range include the lowest recorded SampEn value, suggesting an instance of highly regular $SpO_{2}$ patterns in a particularly fit individual, but it also encompasses the highest value observed in this study. This variation within the group highlights a noteworthy pattern: as fitness levels increase, so does the range of $SpO_{2}$ complexity, with standard deviations correlating positively with fitness levels. This could imply that higher fitness may confer a greater capacity for physiological adaptability, allowing for both higher and lower $SpO_{2}$ variability during strenuous exercise. These findings prompt further investigation into how fitness levels may enhance an individual’s tolerance for $SpO_{2}$ fluctuation and what physiological mechanisms underpin this adaptability. Additionally, we must consider the possibility of technical factors affecting $SpO_{2}$ measurement, which will be rigorously examined in subsequent analyses.

The range of ApEn and SampEn values across athletes of varying fitness levels indicates a possible trend where higher entropy might be linked to more advanced physiological adaptations. Nonetheless, due to significant overlap in entropy values across fitness categories, these measures should not be solely relied upon to assess an athlete’s fitness level, as individual variability plays a significant role.

In summary, the entropy measures of HR and $SpO_{2}$ data across various fitness levels depict a multifaceted landscape of physiological dynamics. Although a tentative trend of increasing entropy with higher fitness levels is observed, the wide distribution and notable presence of outliers caution against a simplistic interpretation of this trend as either linear or nonlinear. The current findings highlight the nuanced and possibly individual-specific responses to fitness, underscoring the significant role of individual physiological variance in interpreting entropy metrics. These complexities present a promising avenue for future research to delve into the distinct physiological attributes or adaptations of highly trained individuals, enriching our understanding of the intricate connections between fitness and physiological entropy.

## 4. Conclusions

This study investigated physiological complexity during incremental exercise among female athletes through the lens of Approximate Entropy (ApEn) and Sample Entropy (SampEn) metrics. Our results illuminate a multifaceted interplay between entropy measures and fitness levels, suggesting an association where higher fitness levels are often, but not exclusively, accompanied by increased entropy in physiological time series data. The observed variations in entropy, especially in the $SpO_{2}$ data, underscore that physiological responses are highly individualized, influenced by a myriad of factors including training regimens, genetics, and other physiological nuances. Consequently, while entropy measures offer valuable insights, they should not be the sole indicators of an athlete’s fitness level.

Our results suggest that individuals with higher fitness levels might experience a wider range of physiological responses, as indicated by the larger standard deviations and CVs for $SpO_{2}$ entropy. Notably, the increased variability in $SpO_{2}$ does not correlate with a lack of cardiorespiratory efficiency. On the contrary, it may be indicative of the body’s proficient oxygen utilization at higher levels of physical demand. The efficient extraction of oxygen, as reflected by the steeper oxyhemoglobin dissociation curves in trained muscles, could account for the observed variations.

This study also underscores the importance of selecting appropriate entropy metrics for analysis. We found differences in the consistency of ApEn and SampEn metrics, which are attributable to their distinct calculations; ApEn includes self-matches, while SampEn does not. We observed that ApEn generally yields higher values than SampEn, which may be attributed to self-matches in its computation. This reinforces the perspective that SampEn might be more reliable for analyzing shorter datasets and highlights the importance of carefully selecting entropy measures in research.

The significant variability and potential measurement artifacts, particularly during intense exercise, highlight the need for a more in-depth examination of the factors contributing to the variation in $SpO_{2}$ entropy levels. Heart rate measurements, being more reliably captured, may offer clearer insights into the physiological impact of fitness. These findings illuminate the importance of entropy measures in evaluating the physiological dynamics related to an athlete’s fitness level and call for more nuanced research and discussion in this domain.

Our findings advocate for the integration of entropy measures into athlete monitoring systems, emphasizing the value of a holistic approach that considers individual variability. Further research is necessary to understand the patterns of entropy observed and to investigate the unexpectedly regular $SpO_{2}$ patterns in high-performing athletes. Delving deeper into the nuances of the relationship between fitness levels and physiological data complexity is crucial for comprehensive insights. This strategy holds promise not only for sports science but also for personalized healthcare, where it could transform health monitoring and diagnostics through the incorporation of advanced biofluidic sensor technology.

## Figures and Tables

**Table 1:** Clinical characteristic of the participants.

Subjects N = 27	$\bar{X}\pm \mathrm{SD}$
Age (years)	22.96±6.19
Size (cm)	163.81±6.90
Weight (kg)	57.24±6.70
BMI (kg/m^2^)	21.31±1.98
$HR_{max}$ (bpm)	189.81±8.54
$VO_{2,max}$ (mL/(kg· min))	48.90±7.62

**Table 2:** Descriptive variables of the population according to physical fitness condition.

Physical Fitness Condition	N	${\mathrm{VO}}_{2,\max}$ ± SD	Min	Max
Excellent (>50 mL/kg/min)	11	55.99 ± 5.83	50.90	66.20
Good (40–50 mL/kg/min)	11	46.75 ± 3.03	41.00	50.00
Medium (30–40 mL/kg/min)	5	38.10 ± 0.55	37.50	38.50

**Table 3:** Comparison of Approximate Entropy (ApEn) and Sample Entropy (SampEn) across fitness levels for heart rate (HR) and blood oxygen saturation ( $SpO_{2}$ ). Values are presented as the mean ± standard deviation (SD) and the coefficient of variation (CV) for m=2 and r=0.2SD .

Fitness Condition	ApEn		SampEn	
	**HR ( $\bar{X}\pm \mathrm{SD}$ ; VC)**	** ${\mathrm{SpO}}_{2}$ ( $\bar{X}\pm \mathrm{SD}$ ; CV)**	**HR ( $\bar{X}\pm \mathrm{SD}$ ; CV)**	** ${\mathrm{SpO}}_{2}$ ( $\bar{X}\pm \mathrm{SD}$ ; CV)**
Medium	0.57±0.13 ; 22.17%	0.96±0.49 ; 46.08%	0.33±0.08 ; 24.25%	0.19±0.04 ; 23.08%
Good	0.56±0.09 ; 16.27%	0.90±0.43 ; 47.56%	0.36±0.12 ; 33.19%	0.22±0.10 ; 44.56%
Excellent	0.60±0.09 ; 15.19%	0.85±0.42 ; 49.46%	0.40±0.15 ; 37.20%	0.22±0.11 ; 48.48%

## Data Availability

The data presented in this study are available on request from the corresponding author.

## Abbreviations

The following abbreviations are used in this manuscript:

ApEn	Approximate Entropy
SampEn	Sample Entropy
$VO_{2,max}$	Maximal oxygen consumption
$SpO_{2}$	Oxygen saturation
HR	Heart rate
PPG	Photoplethysmography
HRV	Heart rate variability
EEG	Entropy of electroencephalogram
HCSC	Hospital Clinico San Carlos
BMI	Body mass index
ECG	Electrocardiographic
SD	Standard deviations
CV	Coefficient of variation
